# Photo-induced enhancement of the power factor of Cu_2_S thermoelectric films

**DOI:** 10.1038/srep16291

**Published:** 2015-11-17

**Authors:** Yanhong Lv, Jikun Chen, Ren-Kui Zheng, Junqiang Song, Tiansong Zhang, Xiaomin Li, Xun Shi, Lidong Chen

**Affiliations:** 1 State Key Laboratory of High Performance Ceramics and Superfine Microstructure, Shanghai Institute of Ceramics, Chinese Academy of Sciences, Shanghai 200050, China; 2 CAS Key Laboratory of Materials for Energy Conversion, Shanghai Institute of Ceramics, Chinese Academy of Sciences, 1295 Dingxi Road, Shanghai 200050, China; 3 Univerisity of Chinese Academy of Sciences, Beijing 100049, China; 4 School of Materials Science and Engineering, University of Science and Technology of Beijing, Beijing, China

## Abstract

Element doping is commonly used to adjust the carrier concentrations in semiconductors such as thermoelectric materials. However, the doping process unavoidably brings in defects or distortions in crystal lattices, which further strongly affects the physical properties of the materials. In this work, high energy photons have been used to activate the carriers in Cu_2_S thermoelectric films. As a result, the carrier concentrations, and the respective electrical conductivity as well as Seebeck coefficient are further changed. The photon-induced electrical transport properties are further analyzed utilizing a Parallel circuit model. Due to the realization of optimized carrier concentrations by photon activation, the power factor of Cu_2_S film is improved more than 900 times as compared with the dark data. As compared to the traditional doping process, the approach using photon activation can realize the tuning of carrier concentrations without affecting crystal lattice. This method provides an opportunity to investigate the intrinsic physical properties of semiconductor materials without involving traditional element doping process that usually brings in additional lattice defects or distortions.

Developing low cost, renewable and environmental friendly technologies for harvesting energy from renewable sources is one of the most important issues nowadays. Thermoelectricity is one of such techniques that can be used as a power generator to convert heat energy to electricity or as a solid state heat pump for active cooling^1^,^2^. The performance of thermoelectric (TE) materials is evaluated using the thermoelectric figure of merit *zT*, which is defined as 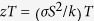, where *σ* the electrical conductivity, *κ* the thermal conductivity, *S* the Seebeck coefficient, and *T* the absolute temperature. Since *σ*, *κ* and *S* are strongly correlated with each other, it is very challenging to realize an independent optimization of these parameters in order to further enhance the value of *zT*. The high thermoelectric performance is usually observed in materials with complex crystal structures for low thermal conductivity^3^ and optimum carrier concentrations^4^ for high power factor (PF = *σS*^2^). Element doping is one of the most common approaches that can be used to adjust the carrier concentrations approaching to the optimum values^5^,^6^, which has been widely used for nearly all the current thermoelectric materials^1^,^2^,^3^,^4^,^5^. Apart from element doping, the carrier concentration can be also adjusted externally by using electrical field^7^, magnetic field^8^ and light radiation^9^,^10^. These approaches have been reported to also realize improved thermoelectric performances. Within the above three external fields, using light radiation is the most simple and easily reachable approach. Meanwhile, it can achieve a strong impact on the tuning of carrier concentrations in semiconductors^9^,^10^.

When photons surpass band gap of materials, photo-induced carriers are generated, leading to enhanced carrier concentrations in semiconductors or insulators^11^. This effect is named as photo doping. The respective changes in Seebeck coefficient and electrical conductivity are defined as photo-Seebeck and photo-conductivity, respectively^9^,^10^. The phenomenon of photo-Seebeck has been observed in many semiconductor materials, such as Ge^12^, Si^13^, GaAs^14^, ZnO^9^ and PbO^10^ for their relatively large band gaps as compared with classic thermoelectric materials^3^,^15^ that usually have narrow band gaps. However, these photo-induced materials usually have low TE performance.

Cu_2–*x*_S compound is a good material for solar energy absorber due to its character of non-toxic, low cost and ideal band gap of 1.2–2.5 eV^16^. High TE performances have been also observed in compounds such as Cu_2–*x*_S^17^,^18^ and Cu_2–*x*_Se^19^,^20^,^21^ due to the liquid-like copper ions. The maximum *zT* in Cu_2−*x*_S is 1.7 at 1000 K, among the top values in thermoelectric materials^17^. Furthermore, experimental data shows the carrier concentrations and Seebeck coefficient at 298 K in Cu_2–*x*_S can be down to 4.8 × 10^18^ cm^−3^ and 0.1 μV/K, respectively, which are quite low values compared to other classic thermoelectric materials^17^. In order to obtain large photo-induced effect, a relatively large band gap and low carrier concentration must be required, as we already known from previous investigations. Therefore, Cu_2–*x*_S compound is an ideal example for investigating the photo-induced thermoelectric properties. It should be noted that thermoelectric thin films have aroused considerable interests within the electronic industry due to its potential applications in rapidly cooling of the microprocessors and sensors^22^,^23^,^24^,^25^. In this work, we investigate the photo-induced electrical properties of Cu_2_S film under ultraviolet light radiation (see Fig. 1). Huge changes in photo-induced Seebeck coefficient and electrical conductivity have been observed. Parallel circuit model is used to analyze the photo-Seebeck and photo-conductivity.

## Result and Discussion

Figure 2 shows the surface morphology of the *as-grown* thin film. Flake-like structures with a small amount of nano-scaled holes are observed. The X-ray maps are applied to analyze the distribution of each element in the films. It is found that all elements are uniformly distributed without element enriching phases. This indicates that the pure Cu_2_S film is obtained in the current study.

Figure 3 shows the ultraviolet (UV) visible-near-IR transmission spectra of Cu_2_S film. It shows a sharp increase at the beginning of the visible region. The optical absorption towards short wave lengths is usually attributed to the transition from valence band to conduction band^26^. The absorption coefficient *α* is calculated by transmittance *M* and film thickness *d* as:


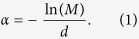


Then the optical energy band gap of Cu_2_S film is estimated by using the classical relation of optical absorption^27^,^28^


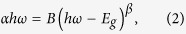


where *B*, *E*_*g*_ and *hω* denote as the band tailing parameter, the optical band gap, and the photon energy, respectively. The value of *β* is taken as *β* = 2, which is a characteristic number for indirect transition which dominates over the optical absorption. In order to calculate the indirect band gap, *(αhω)*^*1/2*^ as a function of photo energy (*E* = *hω*) is plotted in Fig. 3. The curve above 2.5 eV shown in Fig. 3 is linearly extrapolated down to the *x*-axis (*E*) where the absorption coefficient is zero. This gives the absorption energy corresponded to the band gap *E*_*g*_. Using this method, the *E*_*g*_ of our sample is calculated as 1.47 eV, which value is in agreement with the literatures^16^.

**Figure 1:**
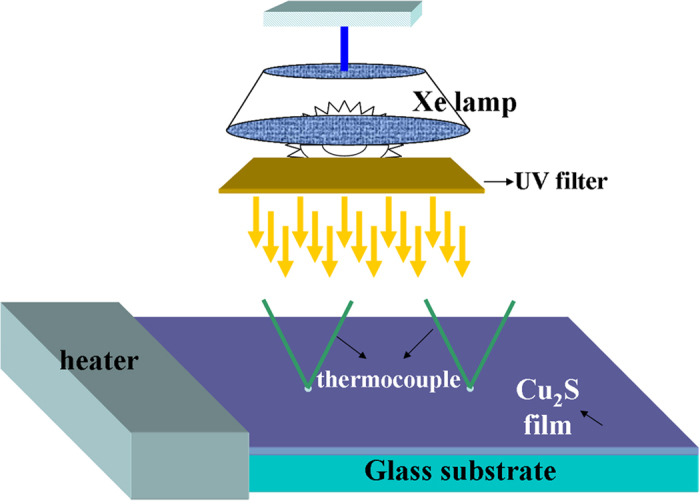
Schematic experimental setup for the measurements of electrical conductivity and Seebeck coefficient under UV light radiation.

**Figure 2:**
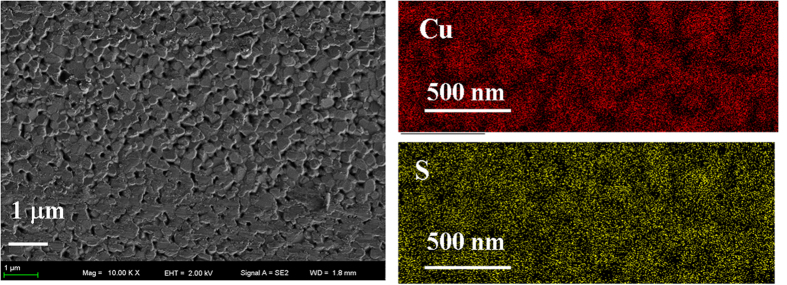
SEM images of the surface morphology (left panel) and x-ray mapping of the elemental distributions (right panel) of the *as-grown* Cu_2_S thin film.

**Figure 3:**
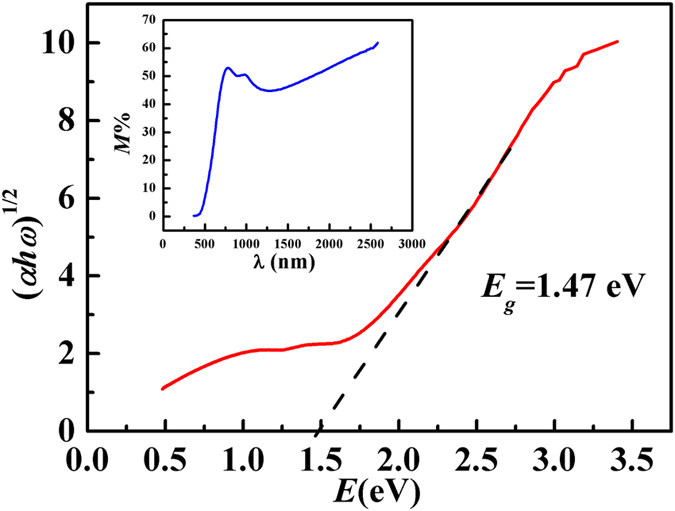
(*αhω*)^*1/2*^ as a function of photon energy *E (E* = *hω*). The inset is the UV-visible-near-IR transmission spectra of Cu_2_S film.

**Figure 4:**
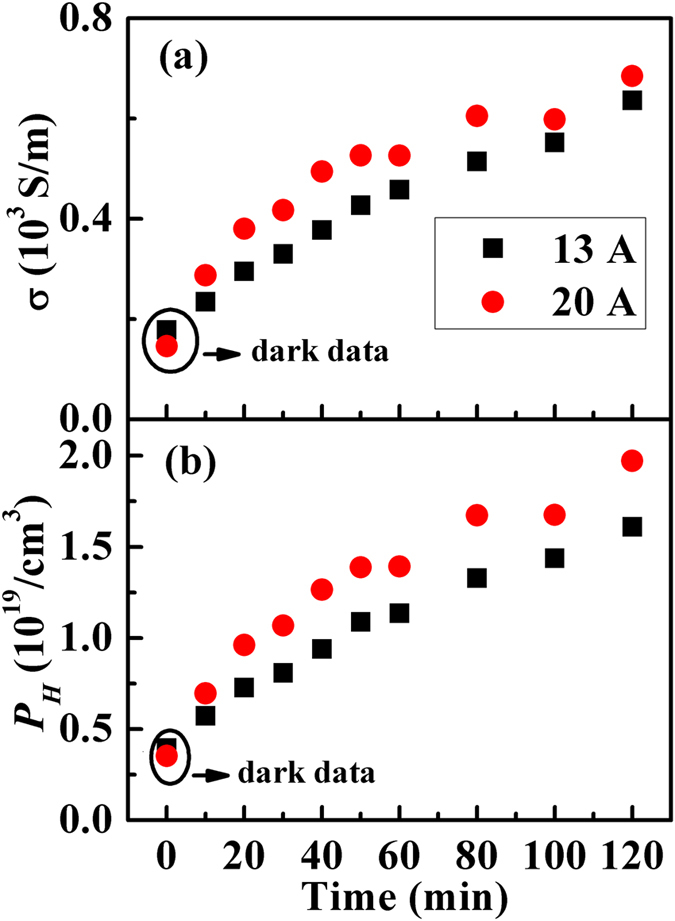
Electrical conductivity and carrier concentration under light radiation. (**a**) Electrical conductivity (*σ*) and (**b**) carrier concentration (*P*_*H*_) at 298 K as a function of light radiation time in Cu_2_S films. The light intensity is controlled by light currents, which are used to mark the samples, the dark square is 13 ampere, and the red cycle is 20 ampere.

In order to study photo-induced electrical transport properties of the Cu_2_S films, the sample was illuminated by UV (*hω* = 3.4 eV) light at 298 K (see Fig. 1). The UV light intensity was controlled by light currents. Figure 4a shows the measured electrical conductivity (*σ*) of the Cu_2_S films at 298 K under light radiation. The dark data of electrical conductivity is around 150 S/m, which is a quite small value as compared to the bulk samples with high *zTs*. An obvious increase in *σ* is observed when increasing light radiation time. The electrical conductivity quickly improved to about 700 S/m in 120 minutes. By increasing the light currents, the electrical conductivity is further increased. The carrier concentration (*P*_*H*_) measured at 298K under light radiation is also shown in Fig. 4b. It displays the same trend. When increasing light currents or light radiation time, the carrier concentration is increased significantly from the order of 10^18^ cm^−3^ to 10^19^ cm^−3^ for more than one order of magnitude. Since ultraviolet (UV) light is used, the increase in the temperature of the sample is less than 7 K when the light radiation time is up to 120 minutes (shown in Fig. S1). Therefore, additional lattice defects in Cu_2_S are not expected to be generated by such a small increase in temperature. Therefore, the enhancement in electrical conductivity and carrier concentration is mainly due to the photo-induced effect.

The electrons and holes are quickly excited by light radiation, and afterwards the electrons are easily trapped by grain boundaries or lattice defects in p-type Cu_2_S thin films. Meanwhile, the excited electrons and holes may also be recombined with each other. The defect-assisted carrier trapping is expected to be faster than the recombination of the excited electrons and holes. Therefore, the measured carrier concentrations are continually increased when increasing the light radiation time. Similar observation and understanding are also reported in a few oxides and sulfides such as PbO^9^, ZnO^10^, PbS^29^.

**Figure 5:**
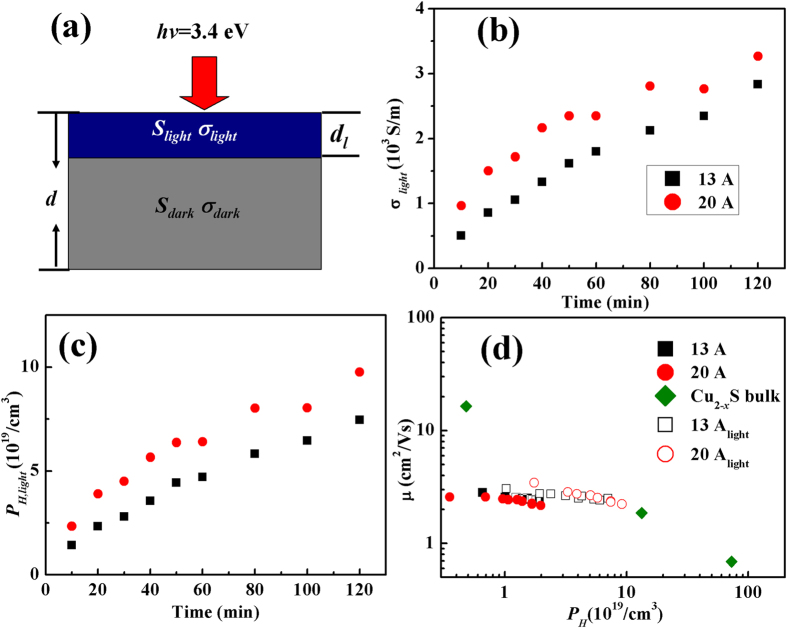
Calculated photo-induced electrical transport properties. (**a**) Schematic illustration of the parallel-circuit model, (**b**) photo-induced electrical conductivity as a function of light radiation time, (**c**) photo-induced carrier concentration as a function of light radiation time, and (**d**) mobility *μ*_*H*_ as a function of carrier concentration. The data measured under light radiation (*μ*_*H*_ ) and calculated in the light penetrating layer (*μ*_*H*,*light*_) have been included for data comparison.

To further characterize the photo-induced transportation properties, the parallel-circuit model^9^,^10^ is used. The model is composed of a thick insulating layer and a thin conducting layer (see Fig. 5a). Under light radiation, the measured Seebeck coefficient (*S*) and electrical conductivity (*σ*) are expressed by


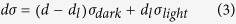


and


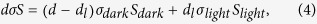


where the subscripts of dark and light represent the electrical transport properties in the pure dark environment and light penetrating layer, respectively. *d* is the sample thickness that is measured by field emission scanning electron microscope (FESEM) with an average value of 200 nm. *d*_*l*_ is the light penetration depth, which is corresponded to the distance that a light can travel in the film from its surface. The *d*_*l*_ is defined as *α*^−1^ 30 and calculated as 33 nm.

In this model, the light penetrating layer and dark layer is assumed to be independent. Therefore, two kinds of carriers exist in the film, which includes the photo-induced carriers in the light penetrating layer and the original charge carriers in the dark layer. The calculated *σ*_*light*_ is plotted in Fig. 5b. Similar to the total electrical conductivity, *σ*_*light*_ also shows a dramatic increase as compared to that the one observed in the dark environment. The photo-induced carrier concentration (*P*_*H,light*_) is calculated by


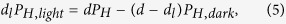


where *P*_*H,dark*_ is the carrier concentration measured in the dark environment and *P*_*H,light*_ is the photo-induced carrier concentration. The maximum value of *P*_*H,light*_ approaches to 1 × 10^20^ cm^−3^, which is about two orders of magnitudes larger than the dark data (see Fig. 5c). In fact, it is very close to the value of the optimized carrier concentration in Cu_2–*x*_S bulk materials^17^. The well enhanced *P*_*H,light*_ strongly suggest we can use only light radiation to realize optimum carrier concentrations in Cu_2_S thin films.

Based on the electrical conductivity and carrier concentration, the carrier mobility (*μ*_*H*_ = *σ/eP*_*H*_) is further calculated and shown in Fig. 5d. Except for the one data in Cu_2_S bulk materials, the *μ*_*H*_ in all other samples including the dark data and the data under light radiation show an almost same trend. A decreasing trend is observed when increasing the carrier concentrations. The *μ*_*H*_ and *μ*_*H,light*_ of Cu_2_S film shows an approximate linear decrease with an increasing carrier concentrations. It is quite interesting to note that the *μ*_*H,light*_ displays almost the same trend as others. For the materials under light radiation, only the electrons or holes are excited by photon energy. The crystal lattice related physical properties are expected to be weakly affected, in particular for the lattice defects. Therefore, the crystal framework should not be changed and the respective changes under light radiation as compared to under dark conditions are considered to come from the different values of carrier concentrations. Therefore, the calculated *μ*_*H,light*_ can be regarded as the carrier concentration-affected carrier mobility in Cu_2_S materials. As we know, this can not be achieved in bulk materials because large amount copper deficiencies must be used to generate high density carrier concentrations. The similar trend shown in Fig. 5d indicates that the slight copper deficiency in Cu_2_S bulk materials does not strongly affect carrier mobility, which is crucial important for realizing high power factors. And this may explain the observed relative high power factors and very high *zTs* in Cu_2–δ_S bulk materials with a certain amount of copper deficiencies.

**Figure 6:**
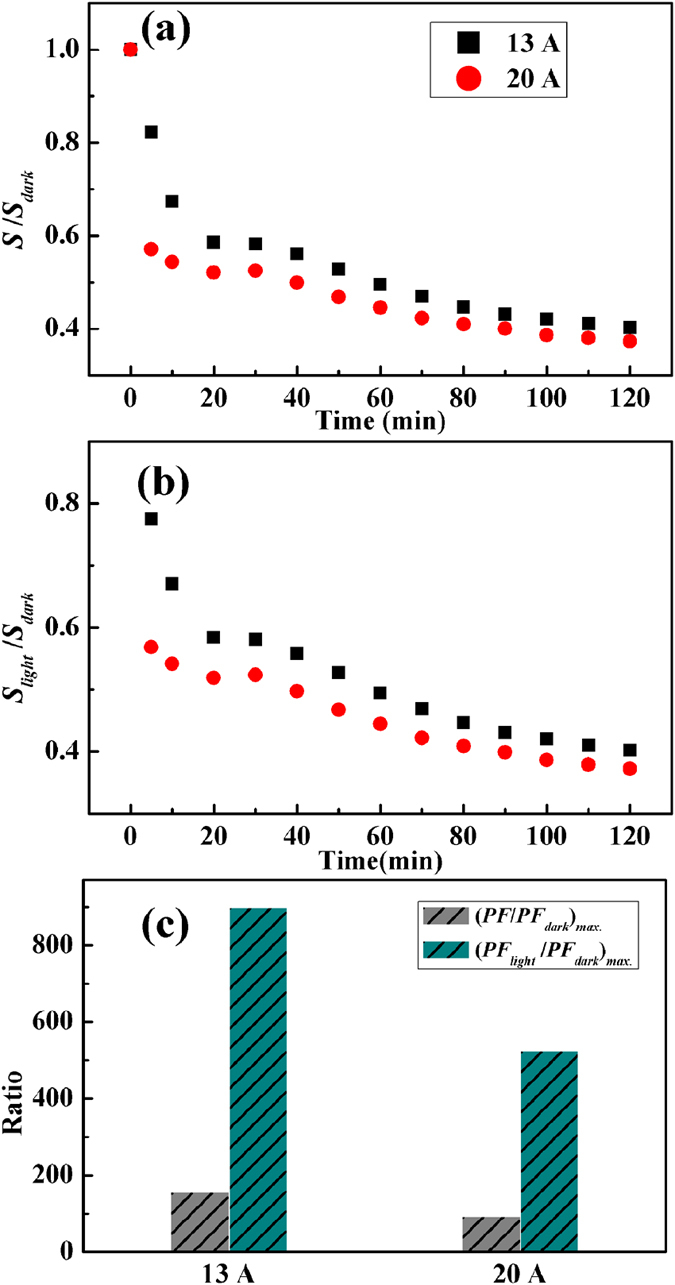
Photo-induced Seebeck coefficient and power factor. (**a**) Relative changes in the total Seebeck coefficient as a function of light radiation time. (**b**) Relative changes in the calculated photo-induced Seebeck coefficient as a function of light radiation time. (**c**) Relative enhanced power factors between the dark data and the power factors under light radiation, and between the dark data and pure photo-induced power factors.

Figure 6 shows the changes in the measured Seebeck coefficient (*S*) of Cu_2_S films under light radiation as well as the changes in the calculated photo-induced Seebeck coefficient in the light penetrating layer (S_*light*_). Without UV light radiation, the films show *p*-type conducting with positive Seebeck coefficients. This is consistent with the Hall measurement, which comes from the nature of copper deficiency in Cu_2_S materials. The dark data of Seebeck coefficient is at a high value about 600 *μ*V/K, since the intrinsic carrier concentration is relatively low. Under light radiation, both *S* and *S*_*light*_ are significantly decreased to about 250 and 210 *μ*V/K, respectively. Similar to the *σ*_*light*_ and *P*_*H,light*_, *S*_*light*_ decreased more as compared to *S*.

Based on the measured and calculated electrical conductivity and Seebeck coefficient, the power factors (*PF*) is further calculated and shown in Fig. 6c. The dark data of *PF* is about 0.016 *μ*W/cmK^2^, due to the low electrical properties. Under light radiation, both *PF* and *PF*_*light*_ significantly increase to about 2.5 and 14.4 *μ*W/cmK^2^, respectively. The photo-induced maximum power factor in the light penetrating layer (*PF*_*light*_) is about 900 times larger than the one observed in dark, due to the enhancing in carrier concentrations towards the optimum value. In contrast to the traditional element doping process, the present adjustment of carrier concentration via photo activation does not affects the crystal lattice. Therefore, this method provides an additional opportunity to investigate the intrinsic physical properties of thermoelectric semiconductors without the contributions from those unexpected defects or impurities when using other approaches or methods.

In summary, the uniform Cu_2_S films have been successfully grown by pulsed laser deposition. The UV-visible-near-IR transmission spectra of Cu_2_S film is used to measure the absorption coefficient. A band gap of 1.47 eV is obtained, while the electrical conductivity, Seebeck coefficient, and carrier concentrations were measured under the UV light radiation with an energy of *hω* = 3.4 eV. Using parallel-circuit model, the pure photo-induced electrical transport properties have been investigated. The photo-induced carrier concentrations can be improved to the order of 10^20^ cm^−3^, two order enhancement compared with the dark data. This leads to a significant enhancement in the electrical conductivity and reduction in the Seebeck coefficient. The electrical transportation properties can be further adjusted by increasing light currents or light radiation time. Due to the optimization of carrier concentration, the photo-induced power factors are enhanced more than 900 times as compared with the dark data without changing the crystal structures. This method provides an additional opportunity to investigate the intrinsic physical properties of thermoelectric semiconductors that excludes the contributions from those unexpected defects or impurities when using other approaches or methods.

## Methods

### Preparation of Cu_2_S thin films

Cu_2_S thin films were grown on glasses by pulsed laser deposition (PLD), using a KrF excimer laser (248 nm, Lamda Physik COMPexPro 201). Film deposition has been carried out on the substrate at 350 °C under argon gas with a pressure of 0.5 Pa, and then the film was subsequently *in situ* annealed at 350 °C for 30 minutes. The laser energy intensity and repetition rate were about 4 J/cm^2^ and 5 Hz, respectively. The chamber base pressure was 2 × 10^−4^ Pa.

### Morphology and optical properties of Cu_2_S thin films

The cross-sectional and surface morphology of the films were examined by field emission scanning electron microscope (FESEM, ZEISS Supra 55). The optical transmittance characteristics were monitored on a Hitachi U-3010 UV-visible-near-IR spectrophotometer with normal incidence from 250 to 2600 nm.

### Electrical transport properties of the Cu_2_S thin films

The electrical transport properties of the films in dark environment and under light radiation were measured using the homemade thermopower measurement systems, which are refitted based on the thermal expansion equipment (Netzsch DIL 402C)^31^ and Accent HL5500 Hall System. In the homemade thermopower measurement systems, two pairs of K-type thermocouples, one heater, and two Au electrodes were packaged into the chamber of the thermal expansion system. The electrical resistivity was measured by the standard four-probe method. The Seebeck was calculated from the slope of the Δ*V vs*. Δ*T* curve upon applying power to a heater and keeping Δ*T* to no more than 2–3 K. In the Accent HL5500 Hall System, the Hall resistance (*R*_*H*_) and electrical resistivity was measured using a four-probe van der Pauw method. It is noted that the sample in the measurements above is prepared at same conditions, but some difference in electrical properties inevitably exited. The ultraviolet (UV) (*hω* = 3.4 eV) light with Xe lamp was employed to run photo-induced electrical transport property measurement. All the measurements are implemented around room temperature (298 K).

## Additional Information

**How to cite this article**: Lv, Y. *et al*. Photo-induced enhancement of the power factor of Cu_2_S thermoelectric films. *Sci. Rep*. **5**, 16291; doi: 10.1038/srep16291 (2015).

## Supplementary Material

Supplementary Information
