# Supplementary material for: Photo-induced enhancement of the power factor of Cu2S thermoelectric films
Source: Sci Rep. 2015 Nov 17;5:16291. doi: 10.1038/srep16291 (PMC4647207; doi:10.1038/srep16291)
Supplement: Supplementary Information [file srep16291-s1.doc]

**Enhanced electrical transport properties in photo-induced Cu_2_S thermoelectric films**

Yanhong Lv,^1,2,3^ Jikun Chen,^2^ Ren-Kui Zheng,^1^ Junqiang Song,^2^ Tiansong Zhang,^2^ Xiaomin Li^1^, Xun Shi,^1,2*^ and Lidong Chen^1,2*^

^1^State Key Laboratory of High Performance Ceramics and Superfine Microstructure, Shanghai Institute of Ceramics, Chinese Academy of Sciences, Shanghai 200050, China,

^2^CAS Key Laboratory of Materials for Energy Conversion, Shanghai Institute of Ceramics, Chinese Academy of Sciences, 1295 Dingxi Road, Shanghai 200050, China,

^3^Univerisity of Chinese Academy of Sciences, Beijing 100049, China

**Corresponding authors:* [*xshi@mail.sic.ac.cn*](mailto:xshi@mail.sic.ac.cn) *and* [*cld@mail.sic.ac.cn*](mailto:cld@mail.sic.ac.cn)

**Supplementary information**


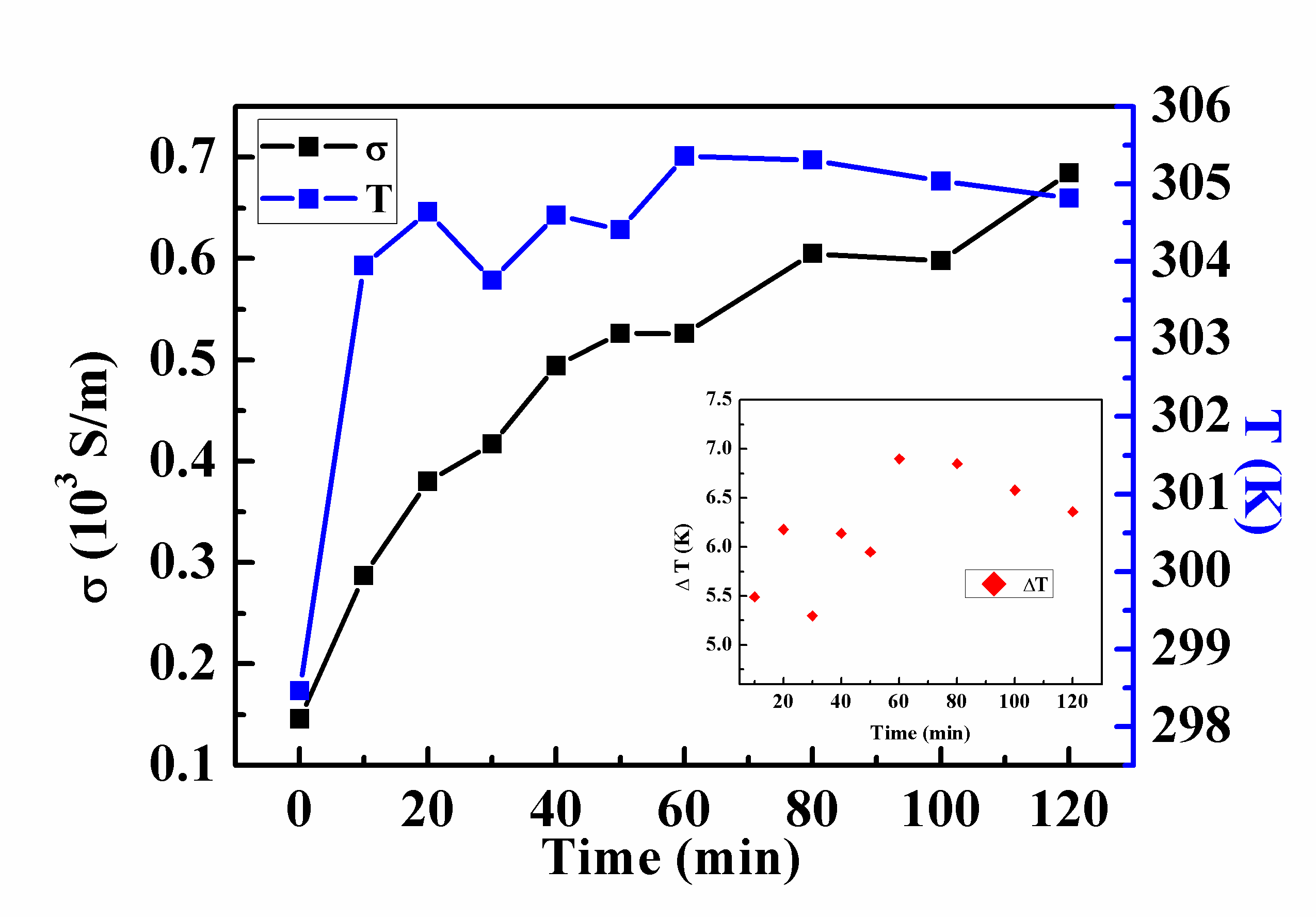


Fig. S1 Electrical conductivity (σ) and sample’s temperature as a function of light radiation time in Cu_2_S films. The insert is the increment of sample’s temperature (*ΔT*) as a function of light radiation time (*ΔT=T-T_0_*, *T* is sample’s temperature and *T_0_* is sample’s dark temperature).
